# Clinical and Inflammatory Characteristics of the Chinese APAC Cough Variant Asthma Cohort

**DOI:** 10.3389/fmed.2021.807385

**Published:** 2022-01-21

**Authors:** Kefang Lai, Wenzhi Zhan, Feng Wu, Yunhui Zhang, Lin Lin, Wen Li, Fang Yi, Ziyu Jiang, Yuanrong Dai, Suyun Li, Jiangtao Lin, Yadong Yuan, Yong Jiang, Chen Qiu, Limin Zhao, Meihua Chen, Zhongmin Qiu, Hu Li, Ruchong Chen, Wei Luo, Jiaxing Xie, Chunxing Guo, Mei Jiang, Xiaohong Yang, Guochao Shi, Dejun Sun, Rongchang Chen, Kian Fan Chung, Huahao Shen, Nanshan Zhong

**Affiliations:** ^1^Department of Pulmonary and Critical Care Medicine, State Key Laboratory of Respiratory Disease, National Clinical Research Center for Respiratory Disease, Guangzhou Institute of Respiratory Health, The First Affiliated Hospital of Guangzhou Medical University, Guangzhou, China; ^2^Department of Pulmonary and Critical Care Medicine, Huizhou The Third People's Hospital, Huizhou, China; ^3^Department of Pulmonary and Critical Care Medicine, The First People's Hospital of Yunnan Province, Kunming, China; ^4^Department of Pulmonary and Critical Care Medicine, Guangdong Provincial Hospital of Chinese Medicine, Guangdong Provincial Academy of Chinese Medical Sciences, The Second Clinical School of Guangzhou University of Chinese Medicine, Guangzhou, China; ^5^Department of Pulmonary and Critical Care Medicine, Key Laboratory of Respiratory Disease of Zhejiang Province, Second Affiliated Hospital of Zhejiang University School of Medicine, Hangzhou, China; ^6^Department of Respiratory and Critical Care Medicine, The Second Affiliated Hospital of Wenzhou Medical University, Wenzhou, China; ^7^Department of Respiratory and Critical Care Medicine, The First Affiliated Hospital of Henan University of Chinese Medicine, Zhengzhou, China; ^8^Department of Pulmonary and Critical Care Medicine, China-Japan Friendship Hospital, Beijing, China; ^9^Department of Pulmonary and Critical Care Medicine, The Second Hospital of Hebei Medical University, Shijiazhuang, China; ^10^Department of Respiratory and Critical Care Medicine, Shenzhen Hospital of Integrated Traditional Chinese and Western Medicine, Shenzhen, China; ^11^Department of Respiratory and Critical Care Medicine, Shenzhen Institute of Respiratory Diseases, Shenzhen People's Hospital, The First Affiliated Hospital of Southern University of Science and Technology, The Second Clinical Medical College of Jinan University, Shenzhen, China; ^12^Department of Respiratory and Critical Care Medicine, Henan Provincial People's Hospital, People's Hospital of Zhengzhou University, Zhengzhou, China; ^13^Department of Pulmonary and Critical Care Medicine, Songshan Lake Central Hospital of Dongguan City, The Third People's Hospital of Dongguan City, Dongguan, China; ^14^Department of Pulmonary and Critical Care Medicine, School of Medicine, Tongji Hospital, Tongji University, Shanghai, China; ^15^Department of Respiratory and Critical Care Medicine, Xinjiang Interstitial Lung Disease Clinical Medicine Research Center, People's Hospital of Xinjiang Uygur Autonomous Region, Urumqi, China; ^16^Department of Pulmonary and Critical Care Medicine, School of Medicine, Ruijin Hospital, Shanghai Jiao Tong University, Beijing, China; ^17^Department of Pulmonary and Critical Care Medicine, The Inner Mongolia Autonomous Region People's Hospital, Hohhot, China; ^18^National Heart and Lung Institute, Imperial College London, Royal Brompton and Harefield Foundation NHS Trust, London, United Kingdom

**Keywords:** cough variant asthma (CVA), classic asthma (CA), airway inflammation, bronchial hyperresponsiveness, cough

## Abstract

**Background:**

The AtyPical Asthma in China (APAC) cohort is a multi-center prospective, observational cohort set-up to investigate the clinical, pathophysiological features, prognosis, and mechanisms of cough variant asthma (CVA).

**Objectives:**

To present the characteristics of newly physician-diagnosed adults with CVA (*n* = 328) compared to mild-moderate classic asthma (CA, *n* = 206).

**Methods and Main Results:**

CVA subjects showed a higher proportion of female (67.1 vs. 55.3%, *P* = 0.0084), abnormal laryngopharyngeal sensations (71 vs. 51%, *p* < 0.0001) than CA, but presented with near normal spirometry and higher methacholine PD20-FEV1 values [4.2 (1, 8.6) vs. 0.8 (0.4, 4.7), *P* < 0.0001]. Lower fractional exhaled nitric oxide (FENO) levels [38.5 (19.8, 72.5) vs. 53. (28.5, 92.2), *P* = 0.0019], blood eosinophil counts [0.2 (0.1, 0.4) vs. 0.3 (0.2, 0.5), *P* = 0.0014], and sputum eosinophils [2.3 (0.3, 8.0) vs. 12.2 (2, 34.5), *p* < 0.0001] were found in CVA. Despite lower total serum IgE levels in CVA, there was similar proportion of atopy in both groups. The prevalence of cough in CA was 86.4%, while CVA reported more severe cough on Visual Analog Scale, Cough Evaluation Test, and Leicester Cough Questionnaire, similar anxiety and depression scores but better asthma control scores as reflected by Asthma Control Test compared to CA. No correlation was found between cough assessment outcomes and sputum eosinophil count, blood eosinophil count, FENO, spirometry variables, or PD20-FEV1.

**Conclusion:**

Cough variant asthma is distinctive from classic asthma in regard to clinical features, lung function, and airway inflammation. Quality of life is badly impaired as well in spite of better asthma control scores.

## Introduction

Bronchial asthma is characterized by wheeze, dyspnea, chest tightness, and cough, and by variable expiratory airflow limitation (1). While cough occurs usually in association with wheeze and dyspnea in patients with classic asthma (CA), it can also present as the sole manifestation of asthma as first described by Corrao and colleagues in cough variant asthma (CVA) (2). CVA has been recognized as specific form of asthma that is usually diagnosed by bronchial hyperresponsiveness (BHR) and/or diurnal variability in lung function (1, 3, 4). CVA has also been identified as one of the most common causes of chronic cough (ranging from 10 to 42%) (5, 6). According to the onset, triggers, severity, airway inflammation or response to treatment, asthma can be divided into different phenotypes, such as early-onset or late-onset, severe asthma, steroid-resistant asthma, or eosinophilic asthma. By contrast, CVA, one of the most common phenotypes of asthma remains ill-understood partly due to the lack of appreciation that an isolated cough may be caused by asthma (1).

Although some studies have shown that CVA shares similarities with CA in terms of BHR and airway eosinophilic inflammation (7, 8), others have reported milder BHR and airway inflammation in CVA (9). Such discrepancies maybe partly due to reports from single centers, their retrospective design, and the small sample size studied. Another consideration is the lack of information on the inflammation accompanying CVA. Cough associated with asthma can be troublesome and can be a prominent symptom, and the therapeutic efficacy of asthma therapies on cough can be variable in both CVA and CA, which can result in potentially significant physical, psychological, and social morbidity (10–12). The discovery of new treatment targets of asthmatic cough and identification of treatable traits suited to individualized treatment have been hindered by a poor understanding of the physiological, pathological, and molecular mechanisms of asthmatic cough.

To address these issues, we set up the AtyPical Asthma in China (APAC) cohort, a multi-center prospective, observational cohort, with the aim of (i) investigating the clinical features, airway inflammation and prognosis of CVA, (ii) identifying multidimensional phenotypes, treatable traits, and new treatment targets of CVA, and (iii) thereby improving the evaluation and treatment strategies of atypical asthma. In this study, with the aim of investigating the distinct characteristics of CVA, we present the cross-sectional assessment of patients with CVA and those suffering from mild-moderate CA from the APAC cohort consisted of analyses of baseline clinical features, lung function, blood, and airway inflammatory measurements.

## Methods

### Study Design and Subjects

This was a multicenter, prospective, observational cohort study for newly physician-diagnosed adult CVA recruited from outpatient clinics of 17 centers located in nine provinces of China. From December 2017 to February 2020, consecutive newly physician-diagnosed adult CVA and CA subjects were recruited at a ratio of 2:1. Eligible subjects underwent a baseline visit with (1) detailed medical history and physical examination recorded in a standard case report frame; (2) completion of Asthma Control Test (ACT), Cough Evaluation Test (CET), Cough Visual Analog Scale (VAS), Leicester Cough Questionnaire (LCQ), Self-rating Anxiety Scale (SAS), and Self-Rating Depression Scale (SDS); (3) performance of spirometry and assessment of variable airflow limitation; and (4) induced sputum test, fractional exhaled nitric oxide (FENO), chest radiograph, and hematological profiles. The control-based asthma management according to the Global Initiative for Asthma (GINA) guidelines and the Chinese Guidelines for Diagnosis and Management of Cough (2015) (4) were used as general principles of treatment, and asthma management was the responsibility of the physicians at the recruitment sites. The participants with CVA were reviewed monthly after enrollment for at least 6 months.

The diagnosis of asthma was made according to Global Initiative for Asthma (GINA) guidelines and Chinese Guidelines for Diagnosis and Management of Cough (2015) (1, 4). CVA was diagnosed on the basis of (1) chronic cough as the sole or predominant symptom lasting for more than 8 weeks, (2) evidence of variable airflow limitation [positive bronchial challenge test (fall in FEV1 from the baseline of ≥ 20% with 12.8 μmol of methacholine or with 7.8 μmol of histamine), or positive bronchodilator reversibility test (increase in FEV1 ≥ 12% and 200 ml from the baseline)], and (3) cough improved with anti-asthmatic therapy during the follow-up (4). A diagnosis of CA was accepted based on variable respiratory symptoms, such as wheeze, shortness of breath, chest tightness and/or cough, and evidence of variable airflow limitation. Exclusion criteria included (1) experiencing respiratory tract infection within the previous 4 weeks; (2) receiving antiasthma medications within the previous 4 weeks, including oral or inhaled corticosteroid, leukotriene receptor antagonist, or antihistamine agents; (3) obvious abnormality of chest imaging; (4) suspected other causes of chronic cough; and (5) pregnancy, breast-feeding, usage of angiotensin converting enzyme inhibitors drug, history of drug or alcohol abuse, other pulmonary disease, or significant comorbidity likely to influence the conduct of the study.

The local ethics committees at each center approved the study. The research was registered on Chinese Clinical Trial Registry (ChiCTR1800014845). All the participants signed informed consent to participate.

### Assessment

The serum levels of total and specific IgE antibodies were measured by ImmunoCap (Phadia AB, Uppsala, Sweden). Specific IgE antibody levels to common allergens, including dust mite (Dermatophagoidespteronyssinus and Dermatophagoidesfarina), cockroach, mold mix (Penicillium, Cladosporium, Aspergillus, Candida, Alternaria and Helminthosporiumcarposaprum), mixed weed pollens (ragweed, mugwort, dandelion, oxeye daisy, and golden rod), and mixed dander (cat, dog, cattle, and horse) were measured. Atopy was defined as at least one positive specific IgE (>0.35 KU/L) to any of these allergens.

Spirometry and bronchial challenge test were performed according to the current ATS/ERS guidelines (13, 14). The provocative cumulative dose of methacholine causing a 20% fall in FEV1 (PD20-FEV1) was used as a measure for BHR.

FENO was measured in accordance with the standard procedure (15). Briefly, the subjects were informed to exhale to the residual air position, and then inhale deeply via a mouthpiece and then exhale with a constant flow (0.05 L/s) for 10 s using NIOX VERO (Aerocrine Company, Sweden).

Sputum was induced and processed as described previously (4). Briefly, sputum was induced with 3% saline. Sputum plugs were selected and mixed with four times its volume of 0.1% dithiothreitol. The cell smear was stained with hematoxylin-eosin. The differential cell count was obtained by counting 400 non-squamous cells.

ACT was used for assessment of asthma control (16). The cough VAS is a 100-mm scale on which the patients indicated the severity of cough. CET is a validated 5-item test to evaluate the full impact of chronic cough with regard to physical, social, and psychological aspects (17). Cough-related quality of life was assessed by LCQ that contained 19 items divided into three domains (physical, psychological, and social) (18). The SAS and SDS were used for general anxiety and depression assessment, respectively.

### Statistical Analysis

Data were expressed as frequency (percentage), mean ± standard deviation or median, and interquartile range [25%, 75%]. Missing data were not imputed. Statistical comparisons between groups were performed with independent sample t-test for normally distributed data, Mann–Whitney U test for skewed data, and Chi-square tests or Fisher's exact test for categorical variables. The correlation of two parameters was tested with Spearman's correlation test and was plotted through the “corrplot” R package. All analyses were conducted using R software Version 3.6.3 (http://CRAN.R-project.org, R Foundation, Vienna, Austria).

## Results

### Demographic Characteristics

**Figure 1:**
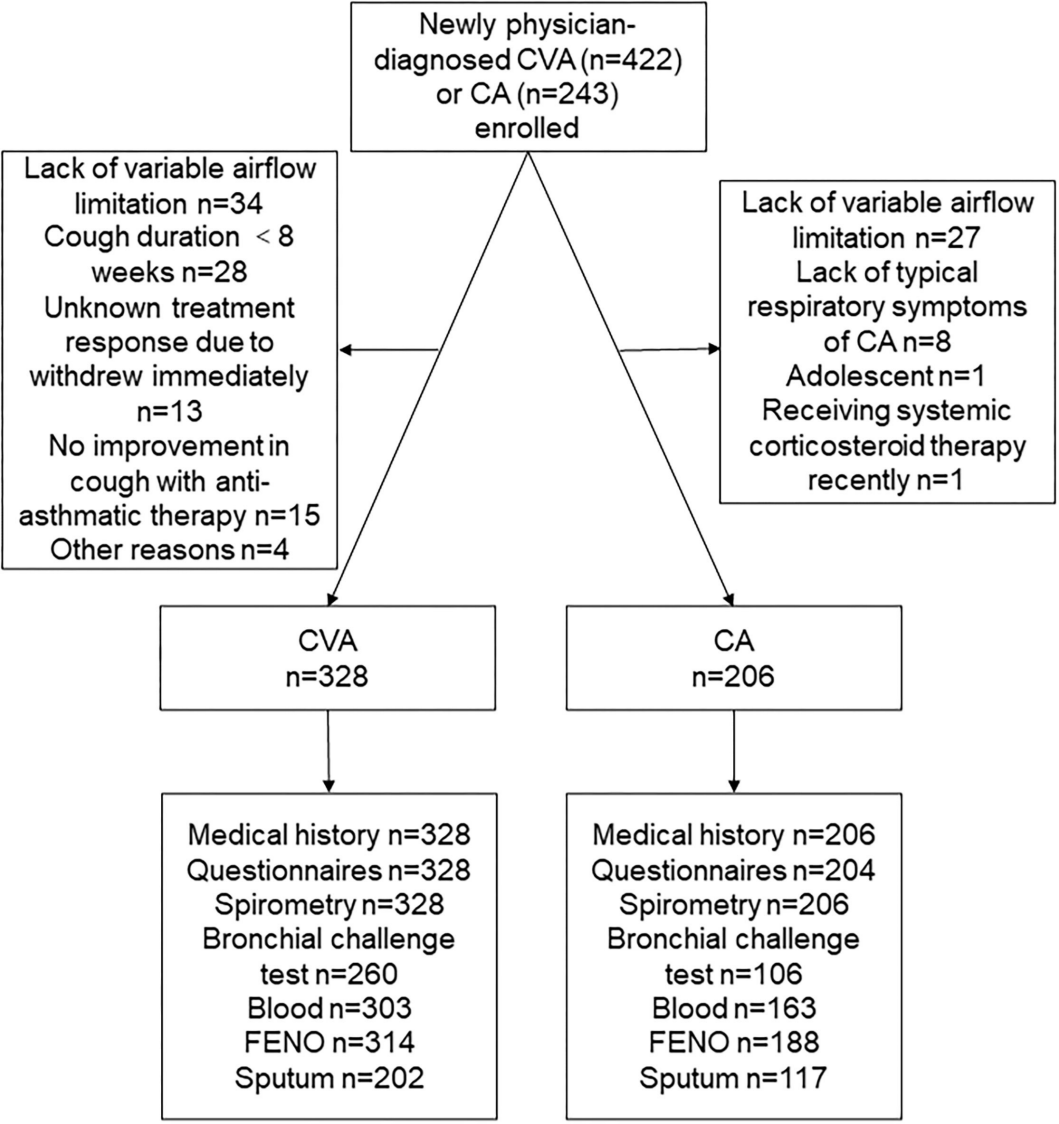
A consort diagram. CVA, cough variant asthma; CA, classic asthma; FENO, fractional exhaled nitric oxide.

From December 2017 to February 2020, 422 newly physician-diagnosed CVA and 243 newly physician-diagnosed CA were recruited. Of the recruited participants, 131 participants were excluded for lacking of variable airflow limitation, cough lasting <8 weeks in physician-diagnosed CVA, withdrew, or other reasons (Figure 1). A total of 328 subjects with CVA and 206 subjects with CA were finally included for the following analysis.

Compared with patients with CA, the proportion of female (67.1 vs. 55.3%, P = 0.0084) was higher and in patients with CVA. There was no significant difference in body mass index, duration of disease, smoking history, educational status, past history of allergies, or family history of asthma between CVA and CA (Table 1).

**Table 1:** Demographic characteristics of subjects.

	**All subjects**	**CVA**	**CA**	***P* value**
Number of subjects	534	328	206	
Age (years)	41.9 ± 12.7	41.0 ± 12.6	43.3 ± 12.9	**0.0419**
Female	334 (62.5%)	220 (67.1%)	114 (55.3%)	**0.0084**
BMI	23.4 ± 3.6	23.4 ± 3.8	23.4 ± 3.1	0.8162
Duration of disease (month)	17.0 [5.0, 60.0]	13.0 [4.0, 48.0]	24.0 [5.0, 68.5]	0.0858
Current-smoker	63 (11.8%)	32 (9.8%)	31 (15.0%)	0.0877
Smoking index (pack-years)	10.0 [3.6, 20.0]	10.0 [4.6, 20.0]	7.5 [3.4, 20.0]	0.4241
**Educational status**				
Primary school or illiteracy	231 (47.0%)	149 (49.2%)	82 (43.4%)	0.4266
High school	192 (39.0%)	112 (37.0%)	80 (42.3%)	
College	69 (14.0%)	42 (13.9%)	27 (14.3%)	
Past history of allergies	136 (25.5%)	80 (24.4%)	56 (27.2%)	0.5357
Food	51 (9.6%)	28 (8.5%)	23 (11.2%)	0.3927
Drug	67 (12.5%)	46 (14.0%)	21 (10.2%)	0.2434
Other	36 (6.7%)	18 (5.5%)	18 (8.7%)	0.2003
Family history of allergic diseases	164 (30.7%)	101 (30.8%)	63 (30.6%)	1.0000
Asthma	85 (15.9%)	46 (14.0%)	39 (18.9%)	0.1653
Eczema	27 (5.1%)	12 (3.7%)	15 (7.3%)	0.0975
Allergic rhinitis	104 (19.5%)	68 (20.7%)	36 (17.5%)	0.4164

*Data are presented as n (percentage), mean ± standard deviation, or median [interquartile range]. CVA, cough variant asthma; CA, classic asthma; BMI, body mass index*.

### Clinical Features and Comorbidities

Cough was a very common symptom in CA with prevalence of up to 86.4%. Compared with CA, the proportion of patients who experienced itchy throat (52.4 vs. 37.4%, P = 0.0009), itching below the throat (13.1 vs. 6.8%, P = 0.0311) or pharyngeal foreign body sensation (26.2 vs. 18.4%, P = 0.0494) was higher in CVA. More patients with CVA (71 vs. 51%, P < 0.0001) experienced abnormal laryngopharyngeal sensations (itchy throat, itching below the throat, pharyngeal foreign body sensation, or frequent throat clearing). There was no significant difference between CA and CVA, in terms of runny nose, postnasal dripping, sneezing, nasal congestion, hyposmia, acid regurgitation, heartburn, or belching (Table 2).

**Table 2:** Clinical features and comorbidities of subjects.

	**All subjects**	**CVA**	**CA**	**P value**
Cough	506 (94.8%)	328 (100.0%)	178 (86.4%)	**<0.0001**
**Concomitant symptoms**				
Runny nose	140 (26.2%)	89 (27.1%)	51 (24.8%)	0.6123
Postnasal dripping	44 (8.2%)	26 (7.9%)	18 (8.7%)	0.8649
Sneezing	219 (41.0%)	131 (39.9%)	88 (42.7%)	0.5856
Nasal itching	142 (26.6%)	76 (23.2%)	66 (32.0%)	**0.0310**
Hyposmia	37 (6.9%)	20 (6.1%)	17 (8.3%)	0.4357
Nasal congestion	154 (28.8%)	89 (27.1%)	65 (31.6%)	0.3177
Itchy throat	249 (46.6%)	172 (52.4%)	77 (37.4%)	**0.0009**
Itching below the throat	57 (10.7%)	43 (13.1%)	14 (6.8%)	**0.0311**
Pharyngeal foreign body sensation	124 (23.2%)	86 (26.2%)	38 (18.4%)	**0.0494**
Frequent throat clearing	106 (19.9%)	71 (21.6%)	35 (17.0%)	0.2295
Abnormal laryngopharyngeal sensations^*^	338 (63.3%)	233 (71.0%)	105 (51.0%)	**<0.0001**
Acid regurgitation	71 (13.3%)	46 (14.0%)	25 (12.1%)	0.6208
Heartburn	17 (3.2%)	9 (2.7%)	8 (3.9%)	0.6334
Belching	31 (5.8%)	20 (6.1%)	11 (5.3%)	0.8615
**Comorbidities**				
Rhinitis	241 (45.1%)	142 (43.3%)	99 (48.1%)	0.3232
Sinusitis	44 (8.2%)	24 (7.3%)	20 (9.7%)	0.4141
Eczema	29 (5.4%)	18 (5.5%)	11 (5.4%)	1.0000
GERD	13 (2.4%)	11 (3.4%)	2 (1.0%)	0.1469
OSA	6 (1.1%)	4 (1.2%)	2 (1.0%)	1.0000
Hypertension	47 (8.9%)	29 (8.8%)	18 (8.9%)	1.0000

*Data are presented as n (percentage). CVA, cough variant asthma; CA, classic asthma; Cough VAS, cough visual analog scale; GERD, gastro-esophageal reflux disease; OSA, obstructive sleep apnoea*.

* *Abnormal pharyngeal sensations included itchy throat, itching below the throat, pharyngeal foreign body sensation, or frequent throat clearing*.

Allergic rhinitis was common in both CVA (43.3%) and CA (48.1%). The prevalence of sinusitis, eczema, gastro-esophageal reflux disease or other comorbidities was <10%, with no significant difference reported in both groups (Table 2).

### Subjective Questionnaire Assessment

The ACT score reflected poor asthma control in both groups, but the ACT score of CA was significantly lower than that of CVA (16.2 ± 3.8 vs. 18.2 ± 3.4, P < 0.0001). Cough of CVA was slightly more severe than that of CA (VAS: 53.7 ± 21.7 vs. 45.5 ± 29.5, P = 0.0004; CET: 13.5 ± 4.1 vs. 12.4 ± 5, P = 0.0067). The cough-specific quality of life reflected by LCQ was lower in CVA, while general anxiety and depression reflected by SAS and SDS scores, respectively, were similar between CVA and CA (Table 3).

**Table 3:** Questionnaire assessment.

	**All subjects**	**CVA**	**CA**	**P value**
ACT	17.4 ± 3.7	18.2 ± 3.4	16.2 ± 3.8	**<0.0001**
ACT scores <20	367 (69.0%)	200 (61.0%)	167 (81.9%)	**<0.0001**
CET	13.1 ± 4.5	13.5 ± 4.1	12.4 ± 5.0	**0.0067**
Cough VAS (mm)	50.7 ± 25.2	53.7 ± 21.7	45.7 ± 29.5	**0.0004**
LCQ-total	14.2 ± 3.6	13.9 ± 3.3	14.8 ± 4.1	**0.0060**
LCQ-physical	4.7 ± 1.1	4.6 ± 1.0	4.8 ± 1.3	**0.0211**
LCQ-psychological	4.6 ± 1.4	4.4 ± 1.3	4.9 ± 1.5	**0.0006**
LCQ-social	5.0 ± 1.4	4.9 ± 1.4	5.1 ± 1.5	0.0653
SAS	38.8 ± 9.8	38.4 ± 9.2	39.6 ± 10.6	0.1980
SDS	38.8 ± 11.6	38.3 ± 11.5	39.4 ± 11.8	0.3252

*Data are presented as mean ± standard deviation or n (percentage). CVA, cough variant asthma; CA, classic asthma; ACT, asthma control test; CET, cough evaluation test; LCQ, leicester cough questionnaire; SAS, self-rating anxiety scale; SDS, self-rating depression scale*.

### Lung Function and Inflammatory Biomarkers

FEV1 (% predicted), FVC (% predicted), and the FEV1/FVC ratio of the patients with CVA were nearly normal and were significantly higher than that in patients with CA (Table 4). The PD20-FEV1 values in CVA were higher than that in CA [4.2 (1, 8.6) vs. 0.8 (0.4, 4.7), *P* < 0.0001].

**Table 4:** Lung function and inflammatory features.

	**All subjects**	**CVA**	**CA**	***P* value**
**Spirometry**				
FEV1 (% predicted)	85.8 ± 17.4	91.1 ± 12.5	77.5 ± 20.6	**<0.0001**
FVC (% predicted)	97.4 ± 14.8	100.0 ± 12.8	93.2 ± 16.8	**<0.0001**
FEV1/FVC (%)	74.4 ± 11.4	77.6 ± 8.5	69.3 ± 13.4	**<0.0001**
MMEF (% predicted)	54.7 ± 22.5	60.0 ± 20.3	45.7 ± 23.2	**<0.0001**
PD20-FEV1 (μmol)	2.8 [0.6, 8.0]	4.2 [1.0, 8.6]	0.8 [0.4, 4.7]	**<0.0001**
FENO (ppb)	44.0 [20.2, 80.8]	38.5 [19.8, 72.5]	53.0 [28.5, 92.2]	**0.0019**
Blood neutrophils (%)	58.4 [52.1, 63.9]	58.7 [52.0, 63.8]	57.5 [52.2, 63.9]	0.8169
Blood-Eos (%)	3.7 [1.9, 6.1]	3.2 [1.6, 5.7]	4.3 [2.4, 7.0]	**0.0039**
Blood-Eos (10^9^)	0.2 [0.1, 0.4]	0.2 [0.1, 0.4]	0.3 [0.2, 0.5]	**0.0014**
Total IgE (KU/L)	128.9 [41.5, 305.6]	106.6 [35.3, 269.5]	160.6 [67.8, 412.8]	**0.0013**
Atopy (any specific IgE ≥ 3.5 (KU/L))	228 (46.0%)	140 (44.4%)	88 (48.6%)	0.4211
**Differential cells in induced sputum**				
Eosinophil (%)	3.4 [0.4, 18.2]	2.3 [0.3, 8.0]	12.2 [2.0, 34.5]	**<0.0001**
Neutrophil (%)	66.5 [36.6, 89.2]	73.1 [45.0, 91.1]	53.0 [28.8, 83.0]	**0.0004**
Macrophage (%)	16.2 [4.2, 36.5]	16.3 [4.2, 37.1]	15.8 [4.5, 35.2]	0.9162
Lymphocyte (%)	0.7 [0.2, 1.4]	0.8 [0.2, 1.5]	0.6 [0.2, 1.2]	0.1733
Elevated Sputum-Eos (≥2.5%)	179 (56.1%)	97 (48.0%)	82 (70.1%)	**0.0002**
Elevated Blood-Eos (≥0.3 ^*^10^9^)	204 (43.8%)	122 (40.3%)	82 (50.3%)	**0.0470**
Elevated FeNO (≥25 ppb)	356 (70.9%)	207 (65.9%)	149 (79.3%)	**0.0021**
Elevation of Sputum-Eos, FENO or Blood-Eos	406 (76.6%)	239 (73.1%)	167 (82.3%)	**0.0203**

*Data are presented as mean ± standard deviation, median [interquartile range] or n (percentage). CVA, cough variant asthma; CA, classic asthma; FEV1, forced expiratory volume in 1 s; FVC, forced vital capacity; MMEF, maximum mid-expiratory flow; FENO, fractional exhaled nitric oxide; Blood-Eos, blood eosinophil count; Sputum-Eos, sputum eosinophil count*.

**Figure 2:**
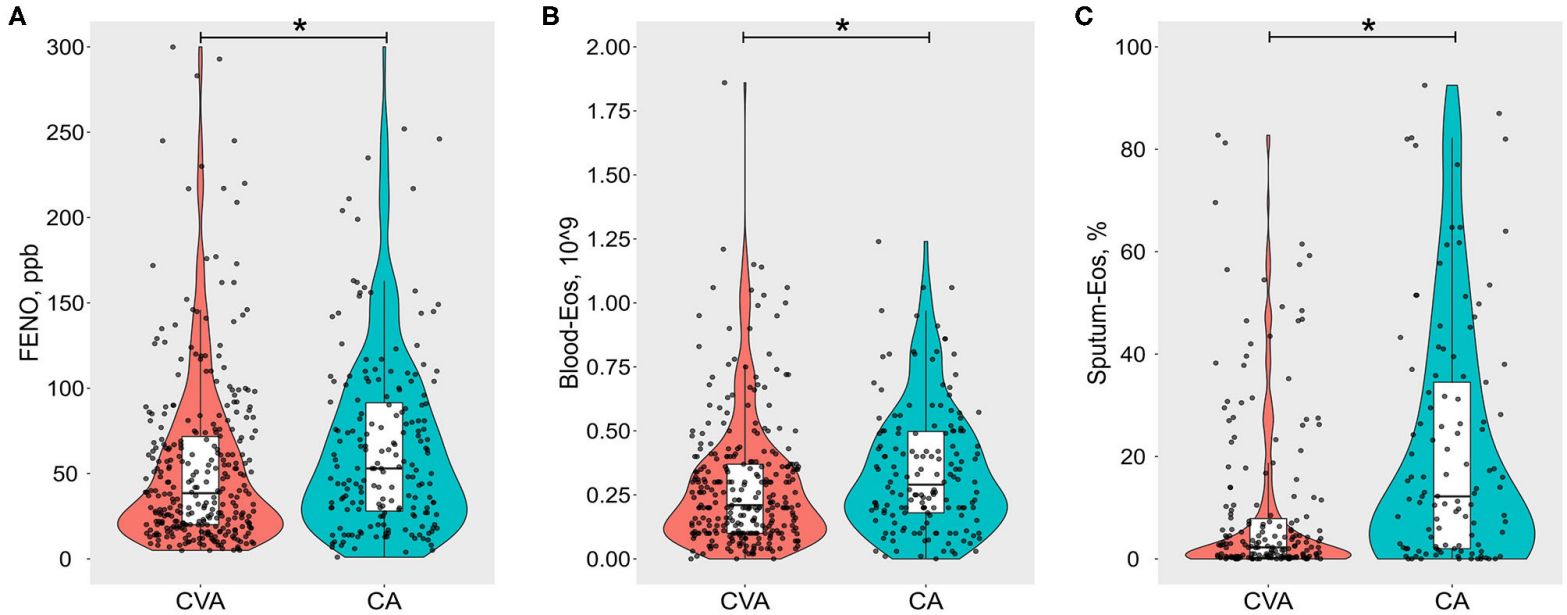
Violin-boxplots of **(A)** fractional exhaled nitric oxide (FENO, ppb), **(B)** blood eosinophil count per 10^9^ (Blood-Eos), and **(C)** sputum eosinophil % (Sputum-Eos) between cough variant asthma (CVA) and classic asthma (CA). The boxes represent median and interquartile range values. Raw data are denoted by black dots, each representing one subject. ^*^Refers to *p* < 0.05.

Compared to CA, there was significantly lower FENO [38.5 (19.8, 72.5) vs. 53 (28.5, 92.2), *P* = 0.0019, Figure 2A], blood eosinophil count [0.2 (0.1, 0.4) vs. 0.3 (0.2, 0.5), *P* = 0.0014, Figure 2B], and sputum eosinophil count [2.3 (0.3, 8.0) vs. 12.2 (2, 34.5), *P* < 0.0001, Figure 2C] in CVA. Similarly, the proportion of sputum eosinophilia (≥ 2.5%), elevated FENO (≥ 25 ppb), or elevated blood eosinophil count (≥0.3 ^*^10^9^) in CVA was significantly lower than that of CA respectively (Table 4). The proportion of elevated FENO, sputum, or blood eosinophils was 73.1% in CVA, which was significantly lower than that in CA (82.3%). Although lower total serum IgE values [106.6 (35.3, 269.5) vs. 160.6 (67.8, 412.8), *P* = 0.0013] were present in CVA, there was a similar proportion of atopy in both groups.

### Medications

At entry, initial treatment was similar between CVA and CA. About 95.7% of CVA and 93.7% of CA were prescribed with inhaled corticosteroids in combination with long-acting beta two agonists. Within the CVA and CA groups, 53.2 and 52.4%, respectively, received leukotriene receptor antagonists, and 14.6 and 8.5%, respectively, received antihistamines therapy.

### Correlation Analysis

**Figure 3:**
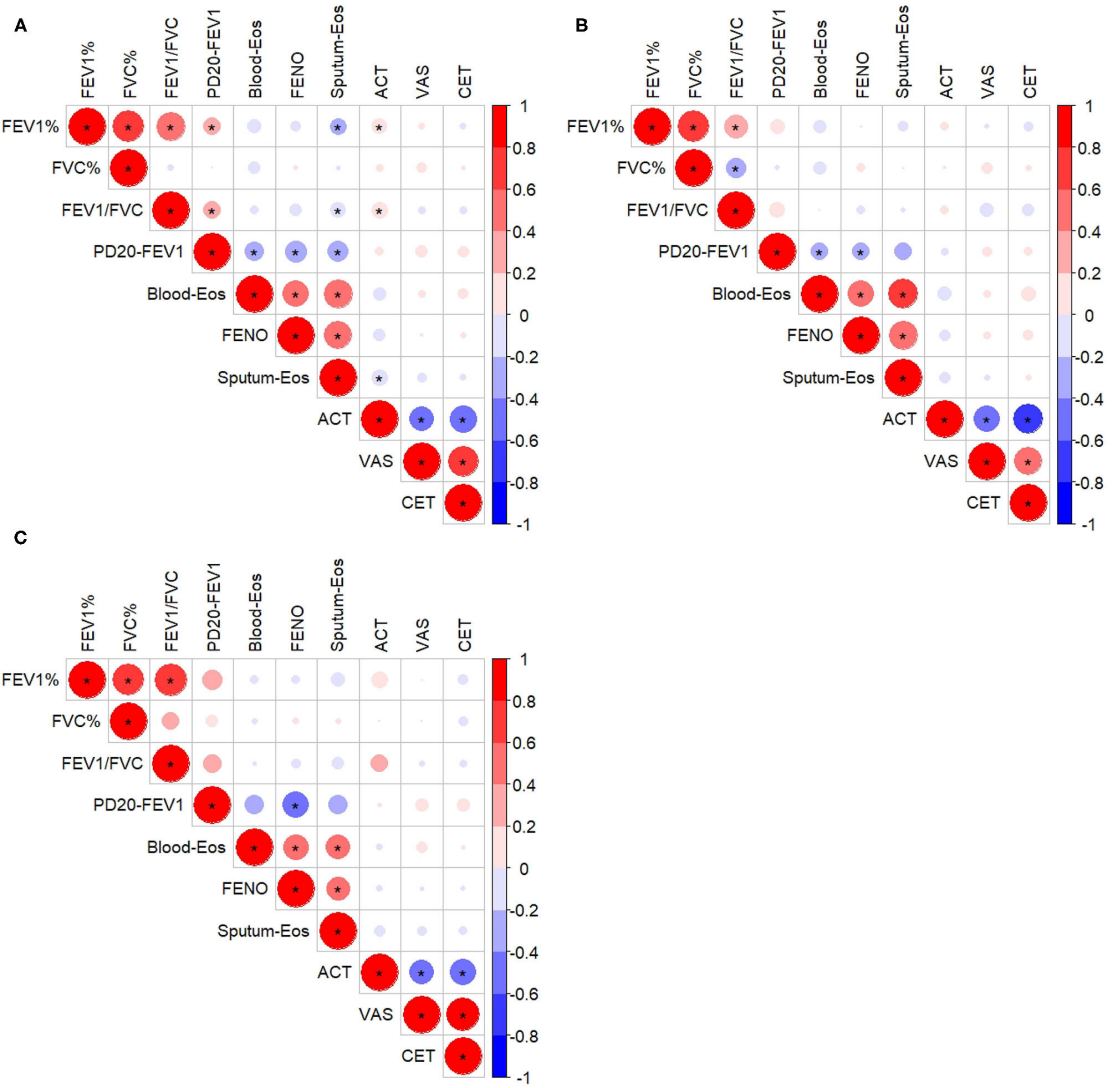
Correlation among inflammatory indexes, lung function, and questionnaire assessments in all the subjects **(A)**, the subjects with cough variant asthma **(B)**, and the subjects with classic asthma **(C)**. ^*^Refers to *p* < 0.05. FEV1%, forced expiratory volume in 1 s, % predicted; FVC, forced vital capacity, % predicted; Blood-Eos, blood eosinophil count; FENO, fractional exhaled nitric oxide; Sputum-Eos, sputum eosinophil count; ACT, asthma control test; VAS, cough visual analog scale; CET, cough evaluation test; PD20-FEV1, provocative cumulative dose of methacholine causing a 20% fall in FEV1. The size of the circle and the intensity of the color represent the correlation coefficient, with the larger size and deeper color indicating a higher correlation coefficient value; a red color indicates a positive correlation, while blue color indicates a negative correlation.

There was no significant correlation between cough assessment outcomes (VAS or CET) and indexes of airway inflammation (sputum eosinophils, blood eosinophils, and FENO), spirometry variables or PD20-FEV1 (Figure 3A). ACT was weakly but significantly correlated with FEV1 (% predicted), the FEV1/FVC ratio, and sputum eosinophils, respectively. PD20-FEV1 showed weak correlation with sputum eosinophil count. However, those correlations were not found when CVA (Figure 3B) or CA (Figure 3C) was analyzed separately.

## Discussion

In this large APAC asthma cohort, we compared patients with newly physician-diagnosed CVA with mild-moderate classic asthma in terms of baseline clinical features, lung function, and inflammatory characteristics. CVA showed female predominance, higher incidence of abnormal laryngeal symptoms, higher ACT scores and slightly more severe cough, while CVA had better lung function, milder BHR, and less severe eosinophilic inflammation as reflected by sputum eosinophil counts, blood eosinophil counts, and FENO, compared with CA. There were comparable proportions of atopy, comorbidities, and accompanying symptoms, as well as a similar degree of general anxiety and depression between CVA and CA.

Asthma and atopic conditions are usually more prevalent in women as from adolescence (19). Compared with CA, while there was a similar proportion of atopy, female predominance in patients with CVA was observed in the current study, as we previously reported (20, 21). The patients with chronic cough also showed female predominance, which might be related to the heightened cough sensitivity reported in females (22). In the current study, we found a similar incidence of rhinitis in CVA and CA. We have recently reported the prevalence of rhinitis in patients with CVA to be 47.1% (21), compared to 43.3% in the current study. There was no significant difference between these two conditions in terms of the incidence of sinusitis, allergic eczema, gastro-esophageal reflux disease, or other comorbidities, indicating that comorbidities may play a little role in the clinical and pathophysiologic differences between CVA and CA.

Sputum eosinophils, blood eosinophils, and FENO are used as biomarkers of airway eosinophilic or Type 2 inflammation (1, 23). We found a high proportion of CVA with sputum and blood eosinophilia, and elevated FENO, as has been previously reported (7–9, 24). But eosinophilic inflammation was milder in CVA compared to CA. However, other studies have reported that there was no significant difference in blood sputum eosinophilia between CA and CVA (7, 8, 24), which may have been due to the small sample size of these studies (12 to 41 subjects per group). Lower FENO in CVA compared to CA has also been reported in other studies (9, 25), together with the proportion of airway eosinophilic inflammation in CVA being significantly lower than that in CA. Nearly one-quarter of patients with newly diagnosed CVA in APAC had non-eosinophilic inflammation according to sputum eosinophil counts, FENO, or blood eosinophil counts. Apart from this large sample size from our multi-center study, another strength of our data is the “natural state” of illness since all our subjects were newly diagnosed CA and CVA who had not received standard anti-asthmatic treatment previously.

There are also conflicting reports regarding BHR with studies showing comparable levels (7, 8, 24), while others showed milder BHR in CVA (9). These differences may be attributed to the different populations and small sample size as well. We found that BHR was milder in CVA than in CA, and we found that sputum eosinophils correlated with BHR. However, the relationship between eosinophils and BHR has been questioned since the anti-IL-5 antibody failed to suppress BHR in spite of a significant reduction in sputum eosinophils (26). There are other potential factors that can contribute to BHR, such as abnormalities of airway smooth muscle, presence of airway remodeling and non-eosinophilic inflammation, and abnormalities in the neural control of the airway caliber (27). However, none of these by themselves fully explain BHR.

Anxiety and depression are common and are comorbidities associated with uncontrolled asthma (28). Compared with CA, the patients with CVA showed comparable general anxiety and depression with slightly worse cough related to quality of life in our data. One study has reported that the patients with CVA were more depressed and anxious than patients with CA (29), suggesting that cough caused similar impact on quality of life in patients with CVA as with CA in spite of minimal or no report of wheeze or dyspnea in CVA. These co-morbid psychological conditions should be considered in the patients with CVA. CVA showed better asthma control status than CA, as assessed by ACT. However, cough is not a symptom that is measured in the ACT questionnaire (16). Increased cough frequency is an indicator of a more severe and difficult to control disease in asthma (30, 31). In addition, the ACT score was only mildly correlated with spirometry or airway inflammation for the whole asthma group, but there was no correlation when CVA or the CA group was analyzed separately, which may lead to an underestimation of the CVA control. Cough frequency reflected asthma control independent of airflow obstruction and inflammation (31). Therefore, the asthma control status of CVA or of cough-predominant asthma should be assessed thoroughly with the inclusion of cough, which is not currently the case.

In this study, the patients with CVA had nearly normal spirometry, which was significantly better compared to the patients with CA. The near-normal spirometric indices with milder bronchial responsiveness are likely factors that account for the absence of wheezing in CVA (32, 33). Cough in the patients with CA was also common. Although CVA presented with more severe cough than in CA, the difference was very modest and was less than the minimal clinically important difference, suggesting that cough was also a bothersome feature of CA, which is not captured on ACT scores. The general assumption underlying the mechanisms of asthmatic cough is that the cough reflex is stimulated by airway inflammatory mediators, mucus, and bronchoconstriction (34). Allergen-induced bronchoconstriction and airway eosinophilia result in increased cough reflex sensitivity associated with an increased cough (35). CVA also showed heightened cough response to bronchoconstriction, and coughing occurred with even mild bronchoconstriction (36). Muscarinic receptor stimulation, bronchoconstriction or bronchodilatation may have no direct effect on the sensitivity of the cough receptors (37). BHR, spirometry, and airway inflammation did not correlate with indices of cough severity, indicating that these parameters may not account for the clinical differences found between CVA and CA.

Neural dysfunction is a feature of asthma as demonstrated by the exaggerated capsaicin-evoked cough responses in asthmatics (38). Furthermore, features of laryngeal hypersensitivity, such as tickle in the throat, throat clearing, and irritation in the throat, are common in patients with chronic cough (39, 40), together with an abnormal sensation in the laryngeal area frequently associated with cough hypersensitivity (40, 41). Compared with CA, a higher proportion of the patients with CVA experience cough-related laryngopharyngeal symptoms, indicating cough hypersensitivity in CVA. In addition, abnormal pharyngeal sensations were distinct features of CVA, irrespective of sex and age (Supplementary E-Table 1). Although we did not measure cough challenge sensitivity, such as capsaicin inhalation challenge, cough hypersensitivity has been reported to be enhanced in the patients with CVA (11, 20), as well as in CA (38). Therefore, cough hypersensitivity may underlie the asthmatic cough. Cough related to cough hypersensitivity is likely resistant to the mainstay treatment consisting of inhaled corticosteroids and/or add on beta 2 agonists, which successfully relieve airway inflammation and airflow obstruction (42). We previously found that heterogeneity of cough hypersensitivity mediated by TRPV1 and TRPA1 was presented in the patients with chronic refractory cough (43). The heterogeneity underlying the development and persistence of chronic cough and cough hypersensitivity begins with the multiple peripheral and central neural pathways capable of eliciting cough and extends to the phenotypic and endotypic presentations that can vary between individual asthmatics with cough (44). Central neuromodulators, such as gabapentin, amitriptyline, and pregabalin, as well as the antagonists of the P2X3 receptor, mainly expressed on the C fibers of the primary afferent sensory nerves, are promising antitussives (45). These neuromodulators and new medications that targeted cough hypersensitivity may improve refractory asthmatic cough. A better understanding of how heterogeneity of cough hypersensitivity is expressed across CA and CVA is needed, which would unveil the pathophysiological and molecular mechanisms of asthmatic cough and facilitate the development of more personalized clinical approaches to manage asthmatic cough.

There are some limitations to our study. First, medical history and symptom assessment were assessed by questionnaires, which may be prone to recall bias. Second, the cough features were only measured by subjective questionnaires with lack of objective measures, such as cough count monitoring or cough reflex sensitivity test. The use of such objective measures of cough would be helpful to understand the asthmatic cough more completely. Third, the success rate in obtaining adequate quality sputum for analysis was only in the 57–62% range, which may lead to bias in the assessment of airway inflammation.

In conclusion, we describe the clinical characteristics of newly physician-diagnosed cough variant asthma in one of the largest cohorts of CVA from a multi-center study in China using standard questionnaires with evaluation of cough, airflow obstruction, bronchial hyperresponsiveness, and biomarkers of inflammation. A higher proportion of females, abnormal pharyngeal symptoms, better lung function, milder bronchial hyperresponsiveness, less severe airway eosinophilic inflammation, together with the absence of wheeze, distinguishes CVA from CA. To enable better understanding of disease mechanisms, it would be important to characterize the cough and cough hypersensitivity that spans across both CA and CVA. In addition, cough is a symptom that should be assessed as part of asthma control.

## Data Availability Statement

The raw data supporting the conclusions of this article will be made available by the authors, without undue reservation.

## Ethics Statement

The studies involving human participants were reviewed and approved by the Institutional Ethics Committee of the First Affiliated Hospital of Guangzhou Medical University (no. 201675). The patients/participants provided their written informed consent to participate in this study.

## Author Contributions

KL, HS, NZ, and RoC: conception and design. WZ, FW, YZ, LL, WLi, FY, ZJ, YD, SL, JL, YY, YJ, CQ, LZ, MC, ZQ, HL, RuC, WL, JX, XY, GS, and DS: recruiting subjects and acquisition of data. MJ, KL, WZ, HS, and NZ: analysis and interpretation. KL, WZ, HS, NZ, and KC: critical review and editing. All authors participated in manuscript writing and editing and read and approved the final manuscript.

## Conflict of Interest

The authors declare that this study received funding from AstraZeneca. The funder was not involved in the study design, collection, analysis, interpretation of data, the writing of this article or the decision to submit it for publication. The authors declare that the research was conducted in the absence of any commercial or financial relationships that could be construed as a potential conflict of interest.

## Publisher's Note

All claims expressed in this article are solely those of the authors and do not necessarily represent those of their affiliated organizations, or those of the publisher, the editors and the reviewers. Any product that may be evaluated in this article, or claim that may be made by its manufacturer, is not guaranteed or endorsed by the publisher.

## Abbreviations

APAC: AtyPical Asthma in China
CA: classic asthma
CVA: cough variant asthma
BHR: bronchial hyperresponsiveness
ACT: asthma control test
CET: cough evaluation test
VAS: visual analog scale
LCQ: leicester cough questionnaire
SAS: self-rating anxiety scale
SDS: self-rating depression scale
FENO: fractional exhaled nitric oxide
FEV1: forced expiratory volume in 1 s
FVC: forced vital capacity
MMEF: maximum mid-expiratory flow
PD20-FEV1: the accumulated provocative dose resulting in a 20% decrease in FEV1
Blood-Eos: blood eosinophils
Sputum-Eos: sputum eosinophils
BMI: body mass index.
