# Supplementary material for: Clinical and Inflammatory Characteristics of the Chinese APAC Cough Variant Asthma Cohort
Source: Front Med (Lausanne). 2022 Jan 21;8:807385. doi: 10.3389/fmed.2021.807385 (PMC8814600; doi:10.3389/fmed.2021.807385)
Supplement: Supplementary file 1 [file Table_1.docx]

| E-table 1. Multiple Logistic Regression Analysis for Cough variant asthma | | | | | | |
| --- | --- | --- | --- | --- | --- | --- |
| Factor | B-value | S.E. | Wald | P value | OR | 95% CI for OR |
| Sex (female) | 0.406 | 0.188 | 4.664 | 0.031 | 1.5 | 1.038-2.168 |
| Age | -0.014 | 0.007 | 3.603 | 0.058 | 0.986 | 0.973-1.000 |
| Abnormal laryngopharyngeal sensations* | 0.817 | 0.192 | 18.195 | <0.001 | 2.264 | 1.555-3.295 |
| Constant | -0.148 | 0.448 | 0.109 | 0.741 | 0.862 |  |
| * Abnormal pharyngeal sensations included itchy throat, itching below the throat, pharyngeal foreign body sensation or frequent throat clearing. | | | | | | |
